# Phylogeny and Cryptic Diversity of *Diopatra* (Onuphidae, Annelida) in the East Atlantic

**DOI:** 10.3390/biology11020327

**Published:** 2022-02-18

**Authors:** Martin M. Hektoen, Endre Willassen, Nataliya Budaeva

**Affiliations:** 1 Department of Natural History, NTNU University Museum, Norwegian University of Science and Technology, 7491 Trondheim, Norway; 2 Åkerblå AS, Nordfrøyveien 413, 7260 Sistranda, Norway; 3 Department of Natural History, University Museum of Bergen, University of Bergen, 5020 Bergen, Norway; endre.willassen@uib.no (E.W.); nataliya.budaeva@uib.no (N.B.)

**Keywords:** Eunicida, species delimitation, morphology, phylogeny, Western Africa, synapomorphy

## Abstract

**Simple Summary:**

*Diopatra* is a genus of marine annelids common in shallow, tropical or subtropical waters. They can occur in great densities and are important ecosystem engineers due to their robust tubes stabilizing sediments. Their large size and striking spiraled branchiae led these worms to be well studied by pioneer taxonomists, and the genus itself was early on well defined. Despite this, delimiting species within *Diopatra* has been difficult due to overlapping morphological characters. In this study we aimed to assess the species diversity of *Diopatra* in West African waters and examine the relationship within the genus using molecular tools. We discovered 17 new species, some of which could not be distinguished from closely related species using only morphology. We also uncovered five well supported clades within *Diopatra*, four of them reinforced by morphological synapomorphies. Extinction rates are increasing in the Anthropocene, and the entire field of conservation biology is directed towards preserving species. A problem facing the field is that it impossible to meaningfully protect what is not known, and there is a fear that species are going extinct before they are discovered. Studies such as this, mapping unknown biodiversity, is imperative in the effort to protect species and biological communities.

**Abstract:**

*Diopatra* Audouin & Milne-Edwards, 1833 is a species rich genus that is common in tropical and subtropical regions. The genus is readily identified by its striking, spiral branchiae, but species identification has historically been challenging due to a high variation in diagnostic characters used. This study aims to reconstruct the phylogeny of *Diopatra* with molecular markers and assess the species diversity of West African *Diopatra* with the species delimitation programs bPTP and BPP. Specimens were collected from Morocco to Angola, and the markers COI, 16S and 28S were sequenced from 76 specimens. The constructed phylogeny retrieved *Diopatra* as monophyletic, as well as five well supported clades within the genus. All clades were defined by morphological characters, some of which have previously not been considered to have high phylogenetic or taxonomical value. Species delimitation analyses recovered 17 new species, several of which were not readily identified morphologically. One species complex comprising between one and 12 species was left unresolved due to incongruence between the species delimitation methods and challenging morphology. Our results indicate that the diversity of *Diopatra* is significantly underestimated, where this regional study near to doubled the number ofknown species from the East Atlantic.

## 1. Introduction

*Diopatra* Audouin & Milne-Edwards, 1833 [1] is monophyletic [2] and the most species-rich genus of the family Onuphidae Kinberg, 1865 [3] with approximately 65 species currently known world-wide [4,5]. *Diopatra* is widely distributed in tropical and subtropical waters and is very common in the intertidal and shallow subtidal zones [6], often playing a key role in ecosystems due to its robust tubes creating habitats and stabilizing the sediments [7,8,9]. The genus has several morphological autapomorphies such as spiral gills, serrated limbate chaetae and complex brush-like tubes [6,10,11]. Although *Diopatra* is very abundant and easily recognizable in many areas, the taxonomy within the genus has been confused throughout history [6,12,13,14,15] due to high intraspecific variation in morphological characters traditionally used in species diagnoses.

Morphology-based phylogeny of the *Diopatra* complex comprising the genera *Diopatra*, *Paradiopatra* Ehlers, 1887 [16], *Paxtonia* Budaeva and Fauchald, 2011 [11], and *Protodiopatra* Budaeva and Fauchald, 2011 [11] was proposed by Budaeva and Fauchald [11] suggesting the monophyly of *Diopatra* which has been later confirmed in a study of phylogenetic relationships among onuphid genera based on two genetic loci [2]. However, a more detailed phylogeny of the genus utilizing molecular data and including a wide array of species has not been previously inferred. *Epidiopatra* Augener, 1918 [17] was erected as a genus closely related to *Diopatra* and differing from the latter only by the absence of peristomial cirri. Only five species were ever described in the genus, four of those from African waters [17,18]. *Epidiopatra* was proposed to be polyphyletic and was subsequently merged with *Diopatra* [11], however, its polyphyly has yet to be confirmed with molecular data.

Currently the East Atlantic fauna encompasses 24 species and subspecies of *Diopatra.* Half of the species were described from shallow western and southern African waters [14,17,18,19,20]. Several recent studies have discussed the diversity of *Diopatra* from the southwestern Europe [21,22,23] and Namibia [24] with the most recent study from the Macaronesian region describing four new species [5]. East Atlantic *Diopatra* species were described mostly based on morphological characters such as: coloration, number of rings on ceratophores, number of whorls of filaments on branchiae, number of denticles on pectinate chaetae, presence or absence of lower postchaetal lobes in anterior parapodia, ventral parapodial lobes, lateral projections on ceratophores, and peristomial cirri. Some morphologically similar species were delimited based on pairwise distance in COI and 16S rDNA sequences between the specimens and using ordination methods on a data matrix of morphological characters [22]. *Diopatra* has previously informally been separated into two main complexes or species groups by morphological characters (e.g., [24,25]: the *Diopatra cuprea* (Bosc, 1802) [26] group, recognized by having numerous denticles on the pectinate chaetae (>15), few rings on the antennophores (<12) and having one postchaetal lobe, and the *Diopatra neapolitana* Delle Chiaje, 1841 [27] group recognized by having fewer denticles on the pectinate chaetae (5–15) and numerous rings on the antennophores (generally > 15).

Cryptic speciation has commonly been reported in many annelid taxa with wide geographical distribution [28,29,30,31,32,33]. Two species of *Diopatra* have traditionally been reported with nearly cosmopolitan distribution: *Diopatra neapolitana* and *D. cuprea*, however many of these records have later been shown to be erroneous [34,35,36]. Due to the historically poor understanding of *Diopatra* taxonomy, it is likely that these cosmopolitan records hide a significant undescribed diversity. Nonetheless, *Diopatra neapolitana* has recently been confirmed with molecular data to occur in the Southwest Atlantic [37], Egypt and Southeast India [38], but it is hypothesized that large species of *Diopatra* are often introduced by anthropogenic means, mostly due to their use as fish bait or aquaculture [37,39,40].

Different species delimitation methods based on gene trees inferred from DNA sequence data can prove invaluable in assessing boundaries between closely related cryptic species [31,41,42]. Poisson Tree Process models (PTP) infer speciation events on gene trees from single locus molecular sequences in terms of number of mutations, and its Bayesian application (bPTP) [43] computes Bayesian support values (PP) to the inferred species. Bayesian Phylogenetics and Phylogeography (BPP) analyze DNA sequence alignments under the multispecies coalescent model (MSC) [44] and can determine whether to collapse or retain input species with a reversible jump Markov Chain Monte Carlo (rjMCMC) method [45,46,47]. The Bayesian support for species computed with this approach is called posterior delimitation probability (PDP).

The aim of this study is to reconstruct the phylogeny of *Diopatra* based on mitochondrial and nuclear markers and to examine the relationships among discovered clades. We also study morphological characters that potentially can be used as synapomorphies for major recovered clades within *Diopatra* and assess the diversity of *Diopatra* in the East Atlantic by applying two sequence-based species delimitation methods: bPTP and BPP.

## 2. Materials and Methods

### 2.1. Material Collection

Material of *Diopatra* was collected by R/V *Dr Fridtjof Nansen* on the West African coast between Morocco and Angola during the Guinea Current Large Marine Ecosystem (GCLME) and the Canary Current Large Marine Ecosystem (CCLME) projects between 2005 and 2012. The sediment samples were obtained using grab, dredge or sledge, washed onboard and fixed either in formalin or 96% ethanol. Preserved samples were sorted to the major taxonomical groups by personnel in the Invertebrate Lab, the Department of Natural History, Bergen. We selected a collection of this material for the study reported in this paper. Material collected in these efforts and used in this study was deposited into the invertebrate collections (ZMBN) of the University Museum of Bergen. We additionally assembled morphological and genetic data on other *Diopatra* from publications and open access web resources, where vouchers are in the Australian Museum, Sydney (AM); the National Museum of Natural History, Smithsonian Institution, Washington, D.C. (USNM); and the Zoological Museum Hamburg (ZMH). All material used in this study is listed in Table S1.

### 2.2. Morphology Study

Specimens were studied using a dissecting stereomicroscope, and temporary slides of parapodia were prepared with glycerol, water or Hydro-matrix^®^(Micro Tech Lab, Graz, Austria), as the mounting medium. Specimens were photographed using a Canon EOS 60D single reflex camera and with a Zeiss Supra 55VP scanning electron microscope. Terminology follows Budaeva & Fauchald [11]. Seven morphological characters and average size (width at 10th chaetiger, excluding parapodia) were scored for 30 putative species and one species complex representing clades obtained in molecular phylogenetic analysis: the presence of an elongated branchial stem defined as branchial stem half as long or longer than filamentous region, presence of lateral projections on the ceratophores, presence of lower postchaetal lobes, hypervariability of denticles on pectinate chaetae within one specimen, defined as a greater variation than 10, where the lowest number of denticles is lower than 15, absence of peristomial cirri, presence of ventral parapodial lobes, and number of rings on lateral ceratophores (Table 1). Six of these characters were binary coded and optimized on the obtained phylogenetic tree. One character, the number of rings on ceratophores, had high degree of intra and inter-specific variation and was not used in the character evolution analysis but provided additional information for morphology-based delimitation hypothesis at species level.

### 2.3. DNA Extraction, PCR Amplification and Sequencing

One hundred and ten specimens in total were selected for genetic analysis, and 76 specimens successfully gave results for at least one marker, covering the entire geographic region sampled by the R/V *Dr Fridtjof Nansen* expeditions. Fragments of cytochrome c oxidase subunit 1 (COI), 16S rDNA and 28S rDNA were selected to be amplified due to previous success with these genes in molecular analyses of *Diopatra* and Onuphidae [2,22]. Primers and amplification protocols used in the study are shown in Table S2. DNA extraction, amplification, and sequencing were done in three different laboratories: Canadian Centre for DNA Barcoding (10 COI sequences, as a part of the Barcode of Life Project “Marine Invertebrates of Western Africa—MIWA”); Macrogen Inc. (14 COI sequences, 19 16S sequences, 38 28S sequences); and the University of Bergen (UiB; 44 COI sequences, 47 16S sequences).

Total genomic DNA was extracted using the QIAGEN DNEasy Blood & Tissue Kit, following the manufacturer’s standard protocol. One to two mm^3^ of tissue was used for the extractions, usually consisting of one or more parapodia for larger specimens, or larger lateral cuts for smaller specimens. Any sign of gut residue and other foreign material was carefully removed from the tissue to reduce the risk of contamination. Extracts were then either sent to Macrogen Inc. for subsequent amplification and sequencing or processed further at UiB DNA-lab and Sequencing facility with the following protocol: 16.35 μL ddH_2_O; 2.5 μL 10× buffer; 2 μL dNTP; 1 μL of each primer; 1 μL DMSO; 0.15 μL TaKaRa taq; 1 μL DNA template. The same PCR mix was used for all amplifications. PCR products were purified using ExoSAP-IT^®^(Thermo Fisher, Oslo, Norway), and sequencing reactions for both strands of the amplified genes were performed using BigDye^®^ Terminator v. 3.1 Cycle Sequencing Kit™, Applied Biosystems(Thermo Fisher, Oslo, Norway). Sequence contigs were assembled in Sequencher v. 4.10.1 (Gene Codes, Ann Arbor, MI, USA). Additional 20 *Diopatra* sequences from 18 specimens were obtained from GenBank, representing species from Australia, the Caribbean, Europe, Japan, and the US. In total, 16 outgroup taxa were selected, 14 from the family Onuphidae and two from Lumbrineridae, some of which were sequenced for this study (Table S1).

### 2.4. Sequence Alignment

Sequences were aligned using the MUSCLE [48] algorithm implemented in MEGA7 [49] using the following settings: 15 gap opening penalty, 6.66 gap extension penalty. The alignment for 16S was further edited by hand in MEGA7. Gblocks v.0.91b [50] was used to eliminate poorly aligned positions in the original alignments. The following parameters were used while masking 16S: minimum number of sequences for a conserved position—49; minimum number of sequences for a flanking position—81; maximum number of contiguous nonconserved positions—8; minimum length of a block—10; allowed gap positions—with half. The following parameters were used while masking 28S: minimum number of sequences for a conserved position—27; minimum number of sequences for a flanking position—45; maximum number of contiguous nonconserved positions—8; minimum length of a block—10; allowed gap positions—with half. The datasets for all three markers were aligned separately and later combined into a single dataset for the analyses. The total molecular dataset consisted of sequences from 110 specimens.

### 2.5. Phylogenetic Analysis

Bayesian analysis was performed in MrBayes v. 3.2.6 [51]. Analyses were run for each locus, as well as for the combined dataset of all three loci. For the combined analysis, five partitions were set up: 16S, 28S, and for each codon position of COI. The most fit evolutionary model for each partition was calculated during the run in MrBayes with the “lset nst=mixed rates=gamma” command. Model parameter values for the three partitions were estimated independently using the “unlink” command. Two independent and simultaneous runs with flat prior probabilities and four chains were run for 12,500,000 generations. Trees were sampled every 1000th generation. The first 25% resulting trees were excluded and the remaining trees were summarized into a majority rule consensus tree with posterior probabilities (PP) indicating the support for each clade. Tracer v. 1.6 [52] was used to examine MCMC sampling statistics and parameter estimates and to verify stationarity with plots of log likelihoods. An effective sample size (ESS) higher than 2000 for the log likelihood and all other parameters when the two runs were combined was considered a good mixing and the results of analyses were accepted. Figtree v. 1.4 was used to visualize the consensus trees [53].

### 2.6. Species Delimitation

Two methods for sequence-based species delimitation were applied, as depending on the dataset, different species delimitation algorithms are likely to give divergent results [54,55]. A Bayesian implementation of the PTP model (bPTP) was used to infer species delimitation from a single locus gene tree. The fundamental assumption is that the number of substitutions between species is significantly higher than the number of substitutions within species [43]. Species delimitation analyses treating COI, 16S and 28S separately, in addition to one analysis treating 16S and COI as one locus were conducted. All analyses were run on the bPTP web server (http://species.h-its.org/; accessed on 18 January 2018) for 500,000 generations, where thinning was set to 100 and burn-in to 0.1. The input tree for all analyses were generated with MrBayes v3.2.6 [51] and outgroups were pruned from the dataset to avoid biases that may arise from distantly related outgroup taxa. Identical sequences were excluded from the analyses as datasets where the number of sampled individuals per species is unbalanced can affect the calculations and might lead to overestimation of the number of potential species [43]. Node support values (posterior delimitation probability; PDP) are strongly correlated to the accuracy of the delimitation (*r* = 0.91; PTP web portal), but no strict cutoff value is usually enforced. We followed the proposed delineations from the analyses which enforced a cutoff at PDP > 0.5 for our dataset.

BPP v. 3.2 [56] was the second method we used for molecular species delimitation. The Joint Bayesian species delimitation and species tree estimation, the A11 analysis [57,58], was used to accommodate for uncertainty in the guide tree. The dataset was split into four parts corresponding with major clades within the genus to avoid model misspecification between distant lineages and the high computational power required for analyzing large datasets in BPP. The gamma priors for the population size parameter (θs) and divergence time (τs) were initially estimated by running the analysis with the species phylogeny given (analysis A00 [44]). In the analysis of clades 1 and 2, the population size parameter (θs) was assigned the gamma prior G(2, 1000), with mean 2/1000 = 0.002, and the divergence time at the root of the species tree (τ0) was assigned the gamma prior G(2, 750). For the analysis of Clade 4, the population size parameters (θs) were assigned the gamma prior G(2, 400), with mean 2/400 = 0.005, and the divergence time at the root of the species tree (τ0) was assigned the gamma prior G(2, 500). The population size and divergence time for Clade 3 was proven difficult to estimate due to many possible species being represented by singletons. As such the priors were set to values representative for the other clades in the genus. θs was assigned the gamma prior G(2, 500) and τ0 the prior G(2, 600). For Clade 5, θs was assigned the prior G(2, 1000) and τ0 the prior G(2, 500). Each analysis was run for 1,010,000 iterations and replicated once to confirm the results did not diverge significantly between the runs. Pairwise distances between and within putative species were calculated in MEGA7 for the COI gene fragment, all gaps and missing data were eliminated from the analysis.

## 3. Results

### 3.1. Phylogenetic Analysis

After being masked in Gblocks, 83.5% of the original sequence length was retained for 16S (430 bp, 235 variable sites and 219 parsimony-informative sites), 92.9% was retained for 28S (637 bp, 255 variable sites and 198 parsimony-informative sites). The COI alignment was 658 bp long, and 327 variable sites and 303 parsimony-informative sites. The final concatenated alignment was 1725 bp long.

**Figure 1:**
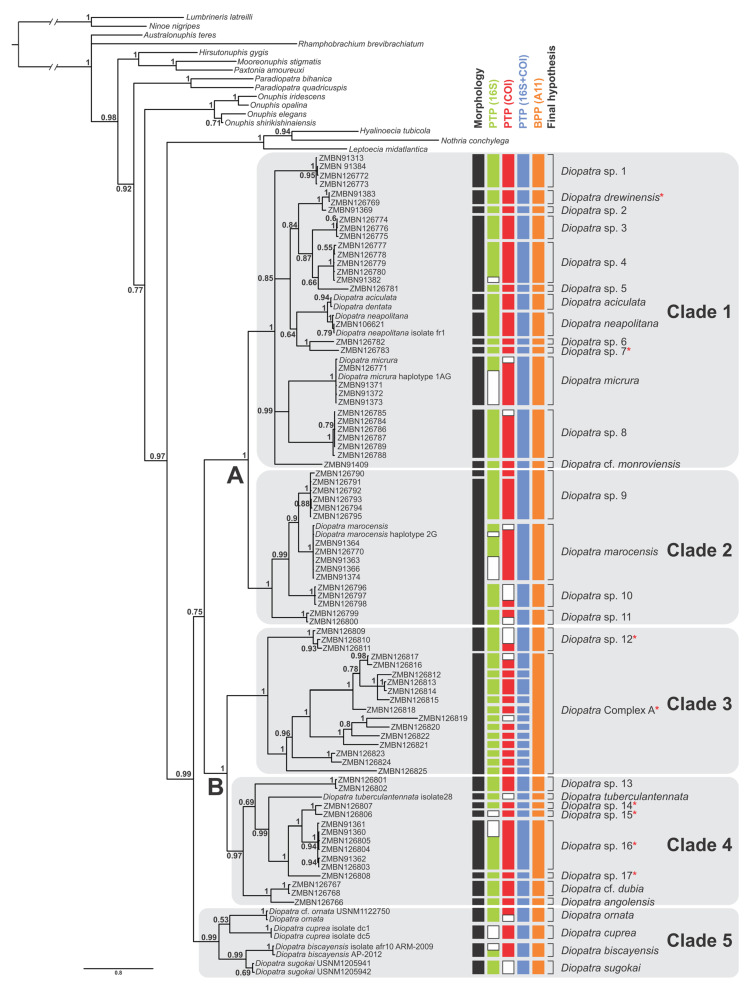
Consensus tree from the Bayesian analysis of the combined COI, 16S and 28S dataset; numbers on nodes indicate Bayesian posterior probabilities; capital letters and clades 1–5 correspond with the clades discussed in the text. Red asterisks indicate species or complexes lacking peristomial cirri, as in the former *Epidiopatra*. Species delimitation results inferred by morphology and DNA-based methods are indicated right to the consensus tree; bPTP was applied separately to the gene trees (COI, 16S and COI+16S treated as a single locus); BPP was based on three loci. White bars indicate missing data.

*Diopatra* was retrieved as monophyletic in the combined analysis of three markers with high support (PP = 0.99) (Figure 1), as well as in the combined analysis of 16S and COI (PP = 0.99) (Figure S1). In the other single locus analyses, *Diopatra* was recovered as monophyletic with insufficient support: 28S (PP = 0.92) (Figure S2) and 16S (PP = 0.58) (Figure S3), while the analysis of single COI marker resulted in paraphyletic *Diopatra* (Figure S4).

The combined analysis resolved five major well supported clades within *Diopatra:* Clades 1–5 (PP between 0.97 and 1.00) (Figure 1). Similar topology with five monophyletic clades was obtained in 16S+COI analysis with each clade having PP = 1 (Figure S1). Analysis based on 28S resulted in monophyletic clades 1 (PP = 0.96) and 2 (PP= 0.57), and Clade 3 (PP = 0.92) nested within paraphyletic Clade 4 (Figure S2). Species from Clade 5 were not included in the 28S analysis. Analysis of single 16S marker resulted in paraphyletic Clade 1 with monophyletic Clade 2 (PP = 1) nested within it. Clades 3, 4 and 5 were monophyletic with PP = 1 (Figure S3). Single locus COI analysis recovered monophyletic Clade 2 (PP = 0.99) nested within paraphyletic Clade 1. Clade 3 was monophyletic but poorly supported (PP = 0.91), while clades 4 and 5 as well as the whole genus *Diopatra* were not recovered as monophyletic with *Paradiopatra* and *Onuphis* Audouin & Milne Edwards, 1833 [1] nested within it (Figure S4).

On the consensus tree (Figure 1), Clade 1 included *Diopatra aciculata* Knox & Cameron, 1971 [59], *Diopatra drewinensis* (Augener, 1918) [17], *Diopatra micrura* Pires et al. 2010 [22], *Diopatra* cf. *monroviensis* Augener, 1918 [17], and *D. neapolitana* as well as four more subclades (each with PP = 1) and four divergent singletons. The relationships within Clade 1 were poorly resolved with several polytomies present. Clade 2 included *Diopatra marocensis* Paxton et al., 1995 [34] and three more well supported subclades (each with PP = 1). Clade 3 combined species previously referred to as *Epidiopatra* and had two well supported sister clades (each with PP = 1), one of which comprised 14 individuals with highly divergent sequences. Clade 4 included *Diopatra angolensis* Kirkegaard, 1988 [20], *Diopatra* cf. *dubia* Day, 1960 [14], *Diopatra tuberculantennata* Budaeva & Fauchald, 2008 [60], two highly supported subclades (PP = 1), and three divergent singletons. Clade 5 combined only species with genetic data obtained from GenBank: *Diopatra biscayensis* Fauchald et al., 2012 [23], *D. cuprea*, *Diopatra ornata* Moore, 1911 [61], and *Diopatra sugokai* Izuka, 1907 [62].

Clades 1 and 2 were sister with high support (PP = 1), noted as Clade A in Figure 1. Similarly, Clades 3 and 4 formed sister relationships with PP = 1, noted as Clade B in Figure 1. The node combining these two higher clades was poorly supported (PP = 0.75) demonstrating the lack of resolution between the largest clades in the *Diopatra* tree.

### 3.2. Species Delimitation

After removing outgroups and identical sequences the PTP was run with 61 sequences for 16S, 67 sequences for COI, 60 sequences for the combined analysis of 16S and COI and 26 sequences for 28S. Thirty-nine putative species were recovered in 16S bPTP analysis with the marker missing from two molecular operational taxonomical units (MOTUs), 40 putative species in COI analysis (marker missing from three MOTUs) and 42 putative species in the analysis treating concatenated 16S and COI as a single locus (present in all MOTUs) (Figure 1). The delimitation results based on COI (Figure S5) were in general better supported than by 16S (Figure S6) and by two combined mitochondrial markers (Figure S7). The bPTP delimitation analysis based on 28S only gave seven putative species (Figure S8), however the MCMC chain for the analysis did not converge even after > 5 attempts (Figure S9), and the dataset was regarded as a poor fit with the bPTP model [43].

The multilocus BPP analysis supported 30 putative species (Figure 1). The main difference in delimitation between the analyses were in Clade 3, where bPTP delimited up to 13 species and BPP supported only two species. In total, we consider there to be 30 distinct species in our dataset based on a combination of the species delimitation results and morphology, with 17 species potentially new to science, and one species complex that we do not delimit further. Morphological differences supported most delimited species, and some of these will be described in an upcoming paper. Delimitation of 15 putative species was congruent in all analyses, including morphology, while 22 species were congruent among all molecular species delimitation methods. This difference was due to the lack of morphological difference between several of the molecular species.

Clade 1

The species delimitation within Clade 1 resulted in 12 species for bPTP 16S analysis, while the other analyses indicated 13 species (Figure 1). The 16S bPTP delimitation received low support (PDP = 0.17) (Figure S6) in splitting *Diopatra neapolitana* and *Diopatra aciculata* Knox & Cameron, 1972 [59], while all other analyses kept the species separate with high support. The specimen in GenBank identified to *Diopatra dentata* Kinberg, 1865 [3] (see Figure 1) has later been confirmed to be *D. aciculata* [38]. The 16S analysis also gave ambiguous results for *Diopatra* sp. 1, with PDP = 0.48 for splitting the species and PDP = 0.51 for keeping it as a single species (Figure S6). The pairwise distance calculation of COI showed that the highest intraspecific variation of the clade was within *Diopatra* sp. 4, with 1.0%, while the lowest interspecific distance was between *D. neapolitana* and *D. aciculata* of 4.3% (Table S3). Distinct morphological differences between most putative species within the clade could be observed, mostly expressed by subtle differences in coloration, but also the presence or absence of peristomial cirri. Clear morphological differences could not be found between *Diopatra* sp. 3, *Diopatra* sp. 4 and *Diopatra* sp. 5, where the characters were largely overlapping. *Diopatra aciculata* and *D. neapolitana* were not examined morphologically in this study, but an in-depth study of the relationship between these species was recently conducted by Elgetany et. al. [38].

Clade 2

The species delimitation results for all analyses received high support for four species, except for bPTP based on COI, which supported five species, by splitting *Diopatra* sp. 9 (PDP = 0.90) into two species (Figure 1 and Figure S5). Specimen ZMBN126790 was divergent morphologically with a distinct brown coloration covering the entire dorsum, not present in any other specimens examined from the clade. Morphological differences between the four delimited species were very subtle and overlapping between the lineages mostly represented by variations in coloration and chaetal morphology and distribution along the body. The greatest COI within-species variation was in *Diopatra* sp. 9, of 1.2%, mainly due to the divergent ZMBN126790 specimen, while the minimum between-species distance was 8.2% between *Diopatra* sp. 9 and *D. marocensis* (Table S3).

Clade 3

Results within Clade 3 were the most divergent between the analyses, with the number of putative species varying between two and 13 (Figure 1). The bPTP analyses based on 16S and combined 16S and COI resulted in 13 putative species, while the COI analysis resulted in 12 putative species. The difference between the bPTP analyses was due to COI not available for the specimen D17. The BPP analysis resulted in two putative species (PP = 0.96 and 0.97). Due to highly divergent results, we only recognize one species, *Diopatra* sp. 12, which was clearly delineated in most analyses, except in the combined 16S and COI bPTP analysis where the species was not well supported (PDP = 0.53) (Figure S7). *Diopatra* sp. 12 clearly differed from the rest of the clade by having a flimsy orange tube with no sediment attached. In addition, it was only found at 500 to 575 m depth, significantly deeper than any other species reported in this study.

The rest of the clade comprised between one and 12 species, but due to low support in the BPP analyses we treat it as a species complex: *Diopatra* Complex A. The specimens in this complex were highly divergent molecularly, with the nearest neighbor of many specimens exceeding 10% (Tables S3 and S4). Although the clade is very distinct morphologically as described earlier, the putative species within the clade were difficult to distinguish. Some minor morphological differences were observed between the specimens, such as coloration, number of teeth on the pectinate chaetae and length of the branchial stems, but since most putative species from the analyses were only represented by a single individual, no information about variation could be obtained.

Clade 4

The delimitation results in Clade 4 varied between seven and eight putative species (Figure 1). Both the bPTP delimitation based on 16S and COI supported seven species due to each marker missing from one species represented by a singleton. The combined 16S and COI analysis recognized eight species. Seven species were delineated in the BPP analysis, in which *Diopatra* sp. 16 and *Diopatra* sp. 17 were combined into one species (PP = 0.99), while all other results recovered them being separate. The COI intraspecific distance varied between 0.6% and 1.6%, while the interspecific distance between nearest neighbors was between 5.3% and 14.3% (Table S3). Morphological differences between all the putative species could be detected through the presence or absence of peristomial cirri and shape and size of lateral projections on ceratophores.

Clade 5

Clade 5 comprised four species: *Diopatra biscayensis*, *D. cuprea*, *D. ornata* and *D. sugokai* (Figure 1). All species received high support from the species delimitation analyses. The morphology was not examined for any species in this clade, however they are clearly distinguished in the literature [23,61,63].

### 3.3. Morphological Traits in Recovered Clades

**Figure 2:**
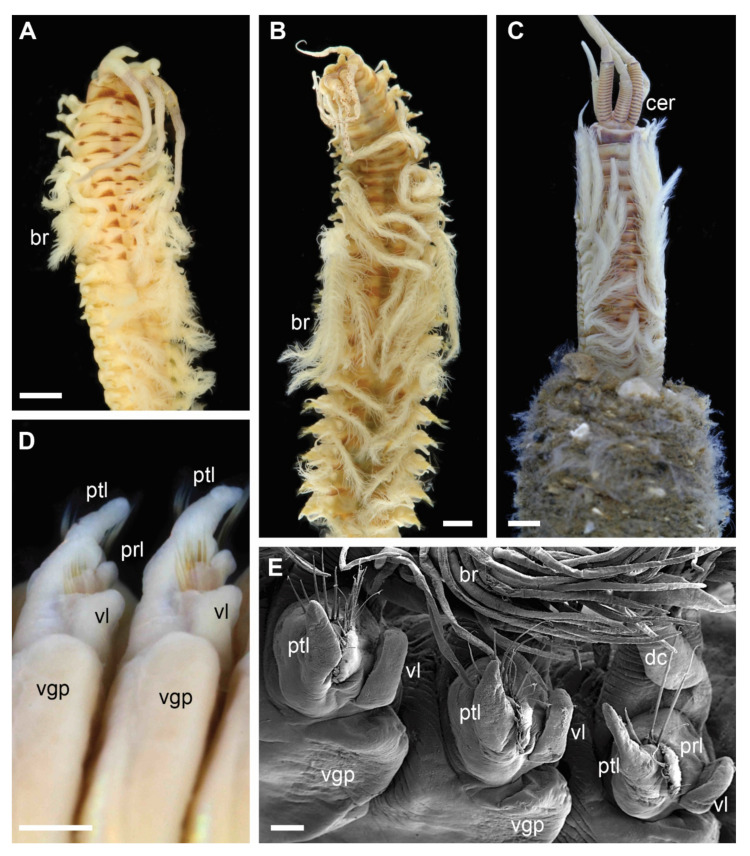
Morphology of *Diopatra* species representing Clade 1. (**A**–**C**)—light microscopy photographs; (**E**)—scanning electron microscopy image. (**A**): *Diopatra* sp. 1 ZMBN91384, anterior fragment showing color pattern, dorsal view; (**B**): *Diopatra* sp. 4 ZMBN91381, anterior fragment showing well-developed branchiae, dorsal view; (**C**): *Diopatra* sp. 2 ZMBN91368, anterior fragment showing numerous rings on ceratophores, dorsal view; (**D**): *Diopatra neapolitana* ZMH P-13819, anterior parapodia showing the presence of ventral lobes, ventral view; (**E**): *Diopatra* sp. 4 ZMBN91380, anterior parapodia showing the presence of ventral lobes, lateral view. br—branchia; cer –ceratophore; dc—dorsal cirrus; prl—prechaetal lobe; ptl—postchaetal lobe; vgp—ventral glandular pad; vl—ventral lobe. Scale bars: (**A**–**D**)—1 mm; (**E**)—100 µm.

**Figure 3:**
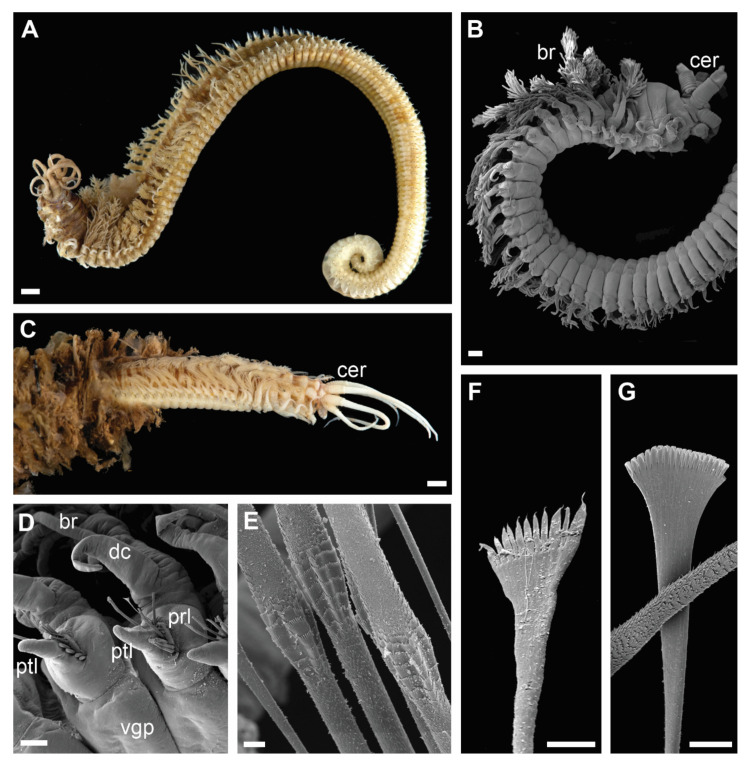
Morphology of *Diopatra* species representing Clade 2. (**A**,**C**)—light microscopy photographs; (**B**,**D**–**F**)—scanning electron microscopy images. (**A**): *Diopatra marocensis*. ZMBN91363, lateral view; (**B**): *Diopatra* sp. 10 ZMBN126797, anterior fragment, lateral view; (**C**): *D. marocensis* ZMBN91378, anterior fragment showing color pattern, lateral view; (**D**): *Diopatra* sp. 10 ZMBN126797, anterior parapodia, lateroventral view; (**E**): The same, limbate chaetae with shelf serration; (**F**): *Diopatra* sp. ZMBN145681, pectinate chaeta from anterior segments; (**G**): The same from posterior segments. br—branchia; cer—ceratophore; dc—dorsal cirrus; prl—prechaetal lobe; ptl—postchaetal lobe; vgp—ventral glandular pad. Scale bars: (**A**,**C**)—1 mm; (**B**)—300 µm; (**D**)—100 µm; (**E**–**G**)—10 µm.

**Figure 4:**
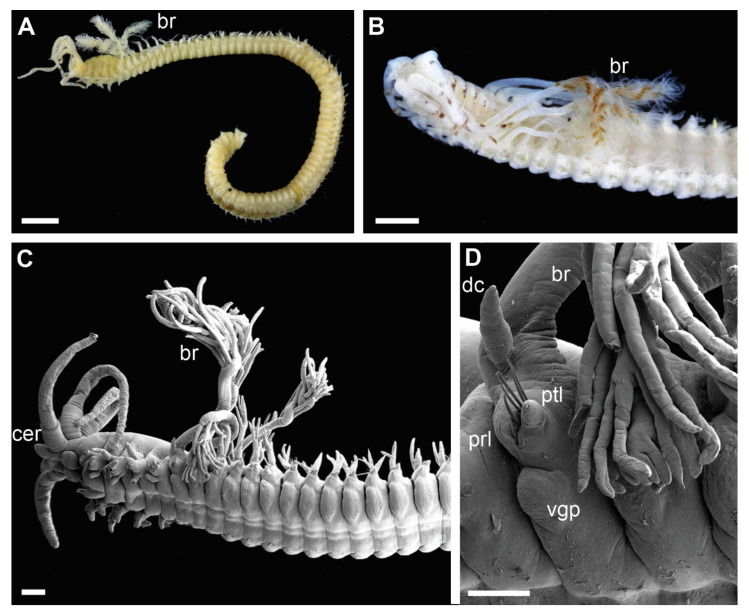
Morphology of *Diopatra* Complex A species representing Clade 3. (**A**,**B**)—light microscopy photographs; (**C**,**D**)—scanning electron microscopy images. (**A**,**B**): Anterior fragment of ZMBN126813 showing first 2–3 pairs of branchiae with long stems; (**C**) anterior fragment of ZMBN145682, lateral view; (**D**): The same as (**C**), parapodium from chaetiger 5, lateral view. br—branchia; cer—ceratophore; dc—dorsal cirrus; prl—prechaetal lobe; ptl—postchaetal lobe; vgp—ventral glandular pad. Scale bars: (**A**,**B**)—1 mm; (**C**)—200 µm; (**D**)—100 µm.

**Figure 5:**
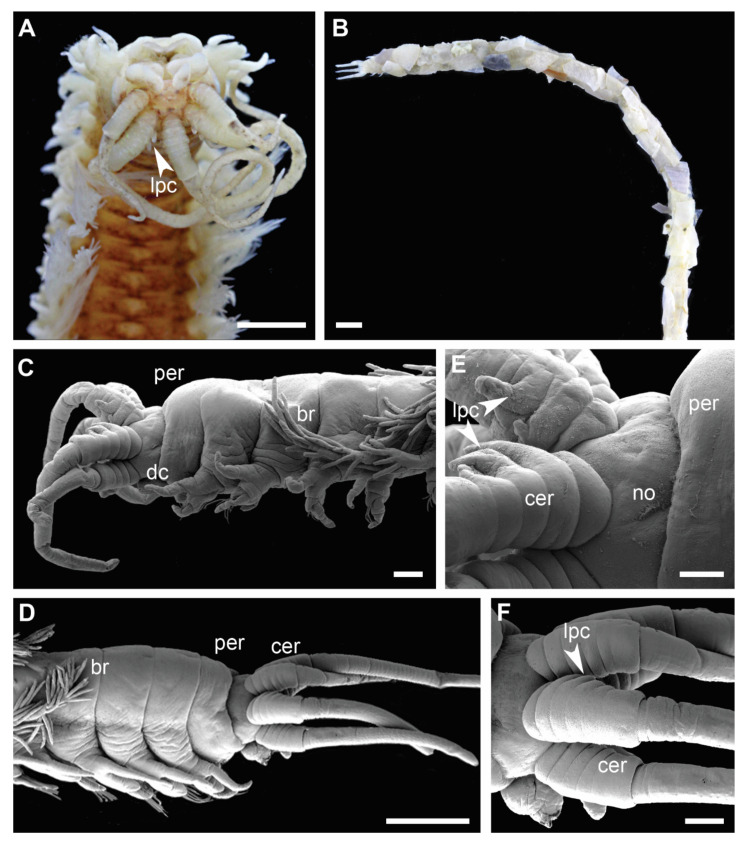
Morphology of *Diopatra* species representing Clade 4. (**A**,**B**)—light microscopy photographs; (**C**–**F**)—scanning electron microscopy images. (**A**): *Diopatra* cf. *dubia* ZMBN145685, anterior fragment with lateral projections on ceratophore rings, dorsal view; (**B**): *Diopatra* sp. 16 ZMBN145684, anterior fragment in a tube, lateral view; (**C**): *Diopatra* sp. 16 ZMBN145683, enlarged, showing peristomium lacking cirri, tube removed; (**D**): *Diopatra* sp. 17 ZMBN126808, anterior fragment lacking peristomial cirri, lateral view; (**E**): *Diopatra* sp. 16 ZMBN145683, prostomium with ceratophores bearing lateral projections, lateral view; (**F**): *Diopatra* sp. 17 ZMBN126808, prostomium with ceratophores bearing lateral projections, lateral view. br—branchiae; cer—ceratophore; dc—dorsal cirrus; lpc—lateral projections of ceratophores; no—nuchal organ; per—peristomium. Scale bars: (**A**)—0.5 mm; (**B**,**D**)—1 mm; (**C**,**F**)—200 µm; (**E**)—100 µm.

**Figure 6:**
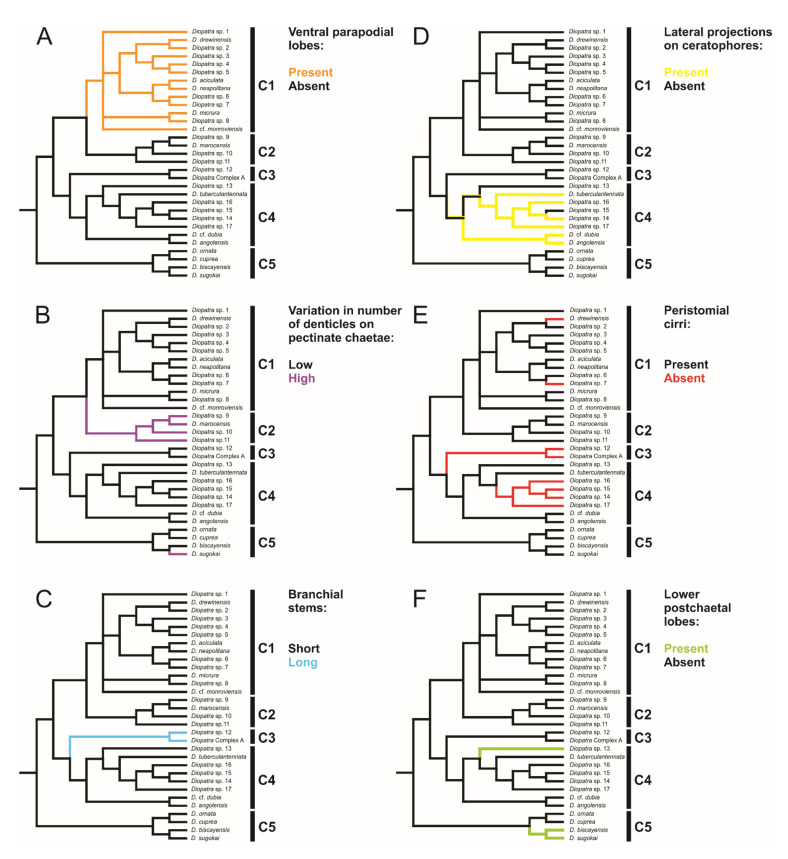
Optimization of six morphological characters described in Table 1 on *Diopatra* species tree. (**A**) Presence or absence of ventral parapodial lobes; (**B**) High or low variation in number of denticles on the pectinate chaetae within a single specimen; (**C**) Short or long branchial stems prior to the filamentous region; (**D**) Presence or absence of lateral projections on ceratophores; (**E**) Presence or absence of peristomial cirri; (**F**) Presence or absence of lower postchaetal lobes. C1–5 represents clades discussed in the text.

Each clade retrieved in the phylogenetic analysis was characterized by morphological characters (Figure 2, Figure 3, Figure 4 and Figure 5). Clade 1 was supported by an exclusive synapomorphy of having ventral parapodial lobes in anterior unmodified segments (Figure 2D,F and Figure 6A). Other shared features within the clade included the high number of rings on the ceratophores (usually more than 15) (Figure 2C), species-specific complex color patterns (Figure 2A–C) and relatively low number (5–15) of denticles on the pectinate chaetae.

Clade 2 consisted of species morphologically similar to *D. marocensis* sharing several characters in chaetal morphology. Hypervariable number of denticles on pectinate chaetae within a single specimen was found in all species of the clade (Figure 3F,G and Figure 6B), but has also been reported in *D. sugokai* from Clade 5 with a variation of 7–30 denticles (Table 1). Another possible synapomorphy of the clade was a strong shelf serration of the limbate chaetae with the serrated part being the widest and tapering into a slender distal blade (Figure 3E). Some individuals in Clade 4 (e.g., *Diopatra* sp. 16) have similar shelf serration, but the distal end of chaetae was wider and the serrated part preceded the widest part of the chaetal distal blade.

Clade 3 was recognized morphologically by having a branchial stem longer than the filamentous region in the first two-three pairs of most developed branchiae (Figure 4B,C and Figure 6C) as well as a very short branchiate region in the midbody segments (Figure 4A–C). This was an exclusive synapomorphy of the clade. One species from Clade 4 (*Diopatra* sp. 13) have branchial stems about the same length or slightly shorter than the filamentous region. In other *Diopatra* species branchial stems are shorter than filamentous regions with many whorls of filaments (Figure 2A–C and Figure 3A–C). Other shared character traits of Clade 3 were the small size of individuals (<1 mm width), subacicular hooks starting around chaetiger 8–9, and the lack of peristomial cirri (Figure 4C).

The presence of lateral projections on ceratophores was a synapomorphy of Clade 4 (Figure 5C–F and Figure 6D), appearing in all but two lineages (*Diopatra* sp. 13 and 15). Two lineages in the clade had tridentate subacicular hooks, a character only reported in one other species of Onuphidae: *Diopatra hektoeni* Paxton & Arias, 2017 [5] also described from the East Atlantic.

Clade 5 was not supported by any apparent morphological synapomorphy but was clearly undersampled in the present study. The lower postchaetal lobe was present in two species from Clade 5 (*D. biscayensis* and *D. sugokai*) but also reported in *Diopatra* sp. 13 from Clade 4.

The absence of peristomial cirri was the main diagnostic character of the former genus *Epidiopatra*. This character trait was present in three of the five clades (Clade 1, Clade 3 and Clade 4) (Figure 4C, Figure 5C–E and Figure 6E) representing a homoplastic synapomorphy of Clade 3 and a subclade nested within Clade 4 combining *Diopatra* spp. 14–17. The presence of lower postchaetal lobes was not found to have any clear phylogenetic importance, appearing in both Clade 4 and 5 (Figure 6F).

### 3.4. Distribution

**Figure 7:**
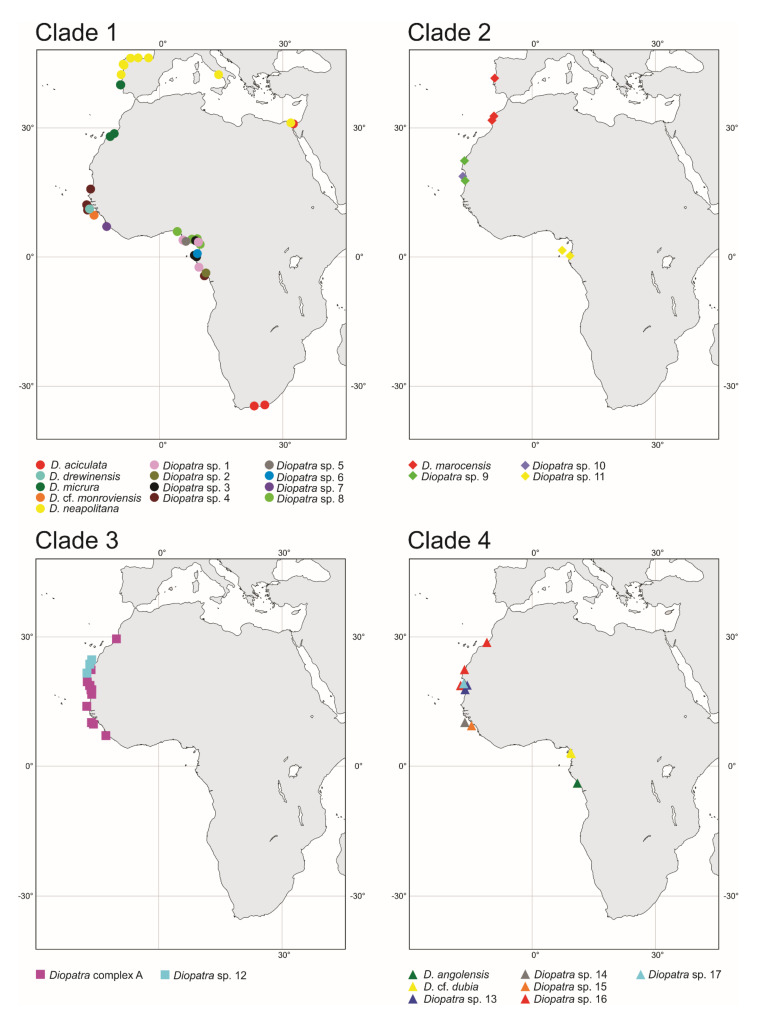
Distribution of *Diopatra* species in East Atlantic waters treated in the present study. The species are grouped by clade numbers (see Figure 1). The distribution of *Diopatra aciculata* and *Diopatra neapolitana* in the study area is based on Elgetany et al. [38].

Most of the recovered putative species displayed a very limited geographical distribution restricted to a single station or several stations within two large zones: Guinea Current Large Marine Ecosystem (GCLME) running from Guinea Bissau to The Democratic Republic of Congo, and the Canary Current Large Marine Ecosystem (CCLME) running from Morocco to Guinea Bissau. The only exception was *Diopatra* sp. 4 which was found along the shoreline of Western Africa from Mauritania to Gabon, occurring in both ecosystems (Figure 7).

## 4. Discussion

### 4.1. Phylogeny

The relationships within *Diopatra* have not yet been well studied by molecular methods. Budaeva & Fauchald [11] published a morphological phylogeny of the *Diopatra* generic complex which included 14 species of *Diopatra* but did not discuss the internal relationships of the genus other than synonymizing *Epidiopatra* with *Diopatra*. Although the present study covers only approximately forty percent of currently known species diversity in the genus, some preliminary conclusions can be outlined. *Diopatra* is well defined as a monophyletic genus by sharing exclusive morphological synapomorphies such as spiral branchiae and serrated limbate chaetae. Our molecular phylogeny supports the monophyletic nature of *Diopatra*, with the exception of the single-locus COI analysis where the genus was retrieved as paraphyletic. Several more characters, i.e., complex tubes, nearly circular nuchal organs, are unique for the genus but are present only in some species. *Diopatra* also displays a unique pattern of chaetal progression and replacement during development, which, however, is understudied, and has only been traced in two species [6,64] and may not reflect the diversity for the whole genus. Species lacking peristomial cirri (Figure 5C,D) occurred in three of the five main clades (Figure 1), supporting the polyphyly of *Epidiopatra* and corroborating the earlier synonymization suggested by Budaeva & Fauchald [11] based on morphological data.

Five major clades were recovered in the analysis with high support. Each of these molecular clades can also be defined by morphological characters, some of which have previously not been considered to have high phylogenetic or taxonomical value. Ventral parapodial lobes (Figure 2D,E) were only present in the 13 species constituting Clade 1, representing an exclusive synapomorphy for this clade. This character is not common outside Eastern Atlantic waters, only being reported in *D. neapolitana*, present in both Western South Atlantic and the Indian Ocean in addition to the East Atlantic, and in *D. aciculata* inhabiting Australian waters, South Africa, and the Red Sea [37,38]. All species in Clade 1 have low to moderate number of denticles on the pectinate chaetae (5–16) and possess ceratophores with numerous rings (in most species 15–20) (Figure 2) with the exception of *Dioptara* sp. 8, which bears only 10–14 rings. However, this is a relatively small-sized species (up to 1.2 mm wide at the 10th chaetiger), and the number of rings in ceratophores is known to be size dependent in Onuphidae (pers. obs.). Paedomorphosis is a commonly reported phenomenon in eunicid annelids [10,65,66], but rarely specifically within *Diopatra*, and few rings can retain in species of presumed paedomorphic origin. The presence of lower parapodial lobes, numerous ceratophore rings and few denticles on pectinate chaetae were used to define the *Diopatra neapolitana* species group, a taxonomically informal unit within the genus *Diopatra* combining similar species [10,24]. The recognition of this group is here supported by molecular data; therefore, we suggest that Clade 1 formally represents *Diopatra neapolitana* species group *sensu* Paxton [24].

Clade 2 encompassed species morphologically similar to *Diopatra marocensis*. The clade can be recognized by a hypervariable number of teeth in pectinate chaetae within the same specimen (Figure 3F,G), found in all lineages of the clade. Number of teeth varies in most other *Diopatra* species as well, but rarely to a high degree. Paxton [10] noted that the number and variation of denticles on the pectinate chaetae, when the number grows larger than 10–15, is usually not useful for species identification, however, the character does seem to have some phylogenetic importance based on our findings. All species in Clade 2 also exhibit limbate chaetae with strong shelf serration (Figure 3E), but this character requires further assessment in other *Diopatra* species using SEM study and thus is only mentioned here. Pectinate chaetae variability and strong serration in limbate chaetae were noted in the original description of *D. marocensis* [34] but were not given significant taxonomic importance.

All specimens recovered in Clade 3 shared three morphological characters: significantly elongated stems and few whorls of filaments in the first 1–2 pairs of branchiae (Figure 4A–C), branchiae abruptly reducing in size posteriorly (Figure 4A–C), and lack of peristomial cirri (Figure 4C). This could be the retention of the juvenile traits, and in fact all specimens in the Clade 3 were small (width < 1 mm at the 10th chaetiger) and also retained other features often characteristic for juvenile specimens such as underdeveloped nuchal organs, unidentate pseudocompound falcigers, and subacicular hooks starting at around chaetiger 9 [5]. A few specimens belonging to this clade were found to be brooding, indicating that they are in fact adults. Therefore, all members of Clade 3 might have undergone paedomorphic evolution leading to miniaturization and underdevelopment of external characters. This would also explain difficulties with species identification and delineation based on morphology as juvenile-like specimens often look alike and lack unique morphological features.

Lateral projections on the ceratophores (Figure 5C–F) were found in all species comprising Clade 4, except in *Diopatra* sp. 13 and 15. Two species in the clade (*Diopatra* sp. 16 and 17) possessed tridentate subacicular hooks, which is unique for *Diopatra* within Onuphidae, and only have been reported from *D. hektoeni* previously [5]. *Diopatra hektoeni* is morphologically close to both *Diopatra* sp. 16 and 17 by lacking peristomial cirri and having lateral projections on ceratophores and is likely closely related to these species.

Clade 5 comprised only species with molecular data obtained from GenBank, and the morphological information from the literature. We could not identify any morphological characters supporting this clade. Two sister species (*D. biscayensis* and *D. sugokai*) had double postchaetal lobe. This is a very conspicuous but rare character within *Diopatra*, previously known only from eight species. Paxton [63] reported intraspecific variation in size of the lower postchaetal lobe in *Diopatra chiliensis* Quatrefages, 1865 [67] ranging from knob-like to subulate. Our results show that a double postchaetal lobe was present in two species from Clade 5 and in *Diopatra* sp. 13 from Clade 4. Therefore, we suggest that the lower postchaetal lobe is a homoplastic character and its development could occur multiple times in the evolution of *Diopatra*.

The *Diopatra cuprea* species group, traditionally used in *Diopatra* taxonomy, has been defined by having ceratophores with less than 15 rings, pectinate chaetae with numerous teeth, single postchaetal lobes, and lacking ventral parapodial lobes [25,34]. Four (rarely three or five) pairs of modified parapodia and bidentate (rarely falcate) pseudocompound (rarely simple) falcigers were also mentioned being characteristic for the *Diopatra cuprea* species group [25], but these character states are present in almost all *Diopatra* species and here are not considered to have any diagnostic value within the genus.

At least six previously described species from our analysis displayed this combination of characters but were recovered in three different clades: *D. cuprea* and *D. ornata* in Clade 5, *D. angolensis*, *D. dubia* and *D. tuberculantennata* in Clade 4 and *D. marocensis* in Clade 2. Furthermore, *D. cuprea* formed a monophyletic clade with two species possessing lower postchaetal lobe. Our results suggest that the presence of pectinate chaetae with numerous teeth and lower postchaetal lobe do not have a clear phylogenetic signal and their value in the system of *Diopatra* was overestimated. Furthermore, the current definition of the *Diopatra cuprea* species group *sensu* Paxton [25] is not valid and requires revision.

We could not identify morphological synapomorphies supporting sister relationships between Clade A and Clade B (Figure 1), however species from Clade A were typically large worms that had well developed external characters, e.g., numerous rings on ceratophores, large branchiae with many filaments, typical brush-like tubes with attached pieces of debris. The species from Clade B were in general of small size and juvenile appearance, with underdeveloped branchiae, short ceratophores, often lacking peristomial cirri and having simple tubes covered with mud, pieces of shells or being parchment-like.

All five recovered clades obtained in the analysis were clearly undersampled, preventing detailed investigation of evolution of morphological characters and relationships between the species in *Diopatra*. Future inclusion of additional species from the genus into analysis will aid in understanding the evolution of the group.

### 4.2. Species Delimitation

Our results increased the number of species reported from the Eastern Atlantic from 24 to 41, in addition to the unresolved species complex that may comprise up to 12 species. These findings clearly show that even a regional faunistic study of *Diopatra* can result in almost doubling the species richness in the area. The novel material covered western African shelf and did not include intertidal localities—the biotope where *Diopatra* species are commonly found in other regions [9,68,69]. Assessing intertidal *Diopatra* diversity may potentially further increase the number of species in western African waters. Recent studies of *Diopatra* from Macaronesia [5] and the Brazilian coast [36] indicated that species diversity in *Diopatra* was highly underestimated. Several regions (e.g., Indian Ocean, South Eastern Asia, Caribbean) are poorly studied and require detailed investigations utilizing an integrative taxonomical approach.

The considerable difference in the number of putative species between bPTP and BPP was mostly due to MOTUs represented by a single individual. Singletons received low support in the BPP analysis, despite significant genetic differences between the lineages. The results in the bPTP analyses were not affected by the number of individuals per lineage. This is especially evident in Clade 3, which despite being genetically very diverse, received low support in the BPP species delimitation analyses and high support in bPTP. There are numerous examples of multispecies coalescent model based species delimitation methods such as BPP overestimating species diversity, eg. [41,70]. Our results do not show this, but rather that BPP might be underestimating the species diversity, such as within the *Diopatra* complex A that the algorithm purports as one species. Two of the assumptions of BPP are the JC69 mutation model and no gene flow between species [57]. The best fitting substitution model for all partitions in our analysis was the general time reversible model (GTR). In addition, hybridization and introgression in metazoan animals as a driving factor in speciation has been shown to be significantly more common than previously thought [71]. Since many of our putative species are sympatric, or close to sympatric, (Figure 7) this could be a factor in our study. BPP is also most suitable for comparing closely related species with divergence < 10% [72], while most COI genetic distances between individuals in Clade 3 exceed this threshold (Tables S3 and S4). The assumption of constant mutation rates may also be violated in some of the divergent lineages. In addition, the number of loci sampled are important for accurate results [73] and only having two loci could impact the results significantly. Nonetheless, BPP supported our hypotheses in most other delineations. While we chose not to distinguish most lineages within Clade 3 to species, we deem it likely that our dataset encompasses several species due to geographic distribution and morphological variation. We expect that with better sampling of Clade 3, the relationships between potential species will be elucidated, and additional distinct species can be formally described.

Two more cases in addition to Clade 3 showed incongruence between the two molecular species delimitation methods. The COI bPTP analysis supported splitting the specimen ZMBN126790 and the rest of *Diopatra.* sp. 9 (Figure S5), while *Diopatra* sp. 9 received high support in all other analyses. As noted earlier, the specimen ZMBN126790 exhibited different morphology than the other specimens representing this species. This was expressed mainly in coloration, while most specimens of this species were cream white, sometimes with small patches of brown coloration dorsally, ZMBN126790 had a distinct brown coloration with no clear pattern. We consider *Diopatra* sp. 9 as one species, however this relationship might be further elucidated by analyzing faster evolving nuclear markers such as ITS1 or 2 and adding more specimens of different color morphotypes into the analysis. The other disagreement between the methods was in delimiting *Diopatra* sp. 16 and 17: BPP supported lumping the species, while the other analyses split them with high support. As *Diopatra* sp. 16 and 17 are not sister species and showed clear morphological differences through the number of chaetigers with pseudocompound falcigers, size, and different shapes of lateral projections on ceratophores, we consider them separate species.

The results based on 28S (Figure S8) were not considered as significant due to lack of convergence between the MCMC chains. This can be partly explained by insufficient genetic variation between and within species preventing from detecting species patterns. With better coverage of the genus, the results might improve [74,75,76].

Cryptic species complexes are common in polychaetous annelids [32], and with the history of taxonomical confusion within *Diopatra* [12,13] and extremely high degree of intraspecific morphological variation, their discovery is not surprising. We recovered three groups of species where clear diagnostic morphological differences were not apparent: *Diopatra* spp. 3, 4 and 5; the entire Clade 2, including *D. marocensis*, *Diopatra* spp. 9, 10 and 11; and all lineages in Clade 3 except *Diopatra* sp. 12 (Figure 1). In the case of *Diopatra* it might be more prudent to refer to these species complexes as pseudocryptic rather than cryptic, as some morphological differences were found. However, these characters were often small, not clearly defined and overlapping between species, making individual-based species identification difficult.

Recent annelid studies have discovered numerous cryptic or pseudocryptic species with restricted geographical ranges and limited depth distribution previously identified as a single widely distributed and eurybathic morphological species [77]. This was corroborated in our study with the most prominent example of *D. marocensis* split into four species with *D. marocensis sensu stricto* found in European waters and Morocco, two possibly sympatric species from Mauritania and West Sahara and one species from equatorial waters of Gabon (Figure 7). Nevertheless, limited geographical and vertical ranges of the recovered *Diopatra* species can be partly explained by the small number of analyzed samples as only localities with specimens with molecular data were used in the study. Inclusion of additional records based on morphology may potentially expand ranges of newly discovered species. We see in the case of *D. aciculata* and *D. neapolitana* that some widely distributed species of *Diopatra* do, in fact, exist [38].

## 5. Conclusions

This study supports the monophyly of *Diopatra* and the polyphyly of *Epidiopatra* presented in Budaeva & Fauchald [11] and begins to elucidate the higher relationships within the genus by uncovering five well supported clades by both molecular and morphological observations. Our results show that the traditional *Diopatra neapolitana* species group is a phylogenetically valid group, whilst the *Diopatra cuprea* species group is not supported. Much time and taxonomic effort has passed since Day debated whether there was one or several species of *Diopatra* worldwide in his 1960 book on South African polychaetes [14]. The present study increases the known species of *Diopatra* in East Atlantic waters from 24 to 41. However, the fact remains that *Diopatra* is morphologically challenging to identify to species, and we uncovered several cryptic species complexes. Our focus in this paper has been on the genetic diversity of *Diopatra* in the studied region, but full morphological descriptions of many of our putative new species are planned for a subsequent study.

## Figures and Tables

**Table 1:** Morphological matrix of eight morphological characters for all species in this study. References for morphological data are shown in Table S1.

Species	Clade	Lateral Projections on Ceratophores	Number of Rings on Antennal Ceratophores	Peristomial Cirri	Lower Postchaetal Lobes	Ventral Parapodial Lobes	Branchial Stems	Number of Denticles on Pectinate Chaetae	Width at 10th Chaetiger, mm
*Diopatra aciculata*	1	Absent	11–19	Present	Absent	Present	Short	5–7	3.5–7.0
*Diopatra drewinensis*	1	Absent	13–20	Absent	Absent	Present	Short	10–13	1.0–3.5
*Diopatra micrura*	1	Absent	12–17	Present	Absent	Present	Short	7–10	0.6–4.5
*Diopatra* cf. *monroviensis*	1	Absent	17–20	Present	Absent	Present	Short	5–7	3.0
*Diopatra neapolitana*	1	Absent	7–16	Present	Absent	Present	Short	5–10	4.0–10.0
*Diopatra* sp. 1	1	Absent	11–17	Present	Absent	Present	Short	8–15	1.5–2.5
*Diopatra* sp. 2	1	Absent	14–17	Present	Absent	Present	Short	9–15	2.0–3.0
*Diopatra* sp. 3	1	Absent	11–16	Present	Absent	Present	Short	10–13	0.8–1.8
*Diopatra* sp. 4	1	Absent	12–20	Present	Absent	Present	Short	10–16	1.5–3.0
*Diopatra* sp. 5	1	Absent	10–13	Present	Absent	Present	Short	8–13	0.8–1.5
*Diopatra* sp. 6	1	Absent	16	Present	Absent	Present	Short	12–15	1.5
*Diopatra* sp. 7	1	Absent	13–14	Absent	Absent	Present	Short	9–13	1.5
*Diopatra* sp. 8	1	Absent	9–14	Present	Absent	Present	Short	5–12	0.6–1.2
*Diopatra marocensis*	2	Absent	6–9	Present	Absent	Absent	Short	12–22	2.0–4.5
*Diopatra* sp. 9	2	Absent	7–11	Present	Absent	Absent	Short	7–18	1.0–2.0
*Diopatra* sp. 10	2	Absent	6–9	Present	Absent	Absent	Short	13–25	1.0–2.0
*Diopatra* sp. 11	2	Absent	7–12	Present	Absent	Absent	Short	10–20	2.0
*Diopatra* Complex A	3	Absent	5	Absent	Absent	Absent	Long	8–15	0.4–0.8
*Diopatra* sp. 12	3	Absent	5	Absent	Absent	Absent	Long	7–12	0.4–0.8
*Diopatra angolensis*	4	Present, knob-like	7–8	Present	Absent	Absent	Short	20–25	1.0
*Diopatra* cf. *dubia*	4	Present, subulate	5	Present	Absent	Absent	Short	13–17	1.0
*Diopatra tuberculantennata*	4	Present, subulate	4–8	Present	Absent	Absent	Short	18–20	0.6–1.3
*Diopatra* sp. 13	4	Absent	6–8	Present	subulate or knob-like	Absent	Short	12–20	1.0–2.5
*Diopatra* sp. 14	4	Present, subulate	5–6	Absent	Absent	Absent	Short	12–16	0.5
*Diopatra* sp. 15	4	Absent	5–6	Absent	Absent	Absent	Short	18–20	0.8
*Diopatra* sp. 16	4	Present, subulate	5–6	Absent	Absent	Absent	Short	13–18	0.5–1.0
*Diopatra* sp. 17	4	Present, knob-like	6	Absent	Absent	Absent	Short	11–18	3.0
*Diopatra biscayensis*	5	Absent	6–10	Present	subulate	Absent	Short	10–20	5.0–8.5
*Diopatra cuprea*	5	Absent	8–12	Present	Absent	Absent	Short	18–25	up to 10
*Diopatra ornata*	5	Absent	8–10	Present	Absent	Absent	Short	>25	3.0
*Diopatra sugokai*	5	Absent	7–12	Present	subulate	Absent	Short	7–30	10.0

## Data Availability

All physical specimens used in this study are kept in the collections (ZMBN) of University Museum of Bergen, Norway. All sequences have been submitted to GenBank and BOLD (see Table S1 for accession numbers and BOLD process IDs).

## Supplementary Materials

The following supporting information can be downloaded at: https://www.mdpi.com/article/10.3390/biology11020327/s1, Figure S1: Consensus tree from the Bayesian analysis of the combined COI and 16S dataset, Figure S2: Consensus tree from the Bayesian analysis of 28S dataset, Figure S3: Consensus tree from the Bayesian analysis of 16S dataset, Figure S4: Consensus tree from the Bayesian analysis of COI dataset, Figure S5: bPTP species delimitation tree built based on COI dataset, Figure S6: bPTP species delimitation tree built based on 16S dataset, Figure S7: bPTP species delimitation tree built based on the combined COI and 16S dataset, Figure S8: bPTP species delimitation tree built based on 28S dataset, Figure S9: Posterior trace plot showing lack of convergence in MCMC run in bPTP analysis of 28S dataset, Table S1: List of specimens used in this study with voucher numbers, accession numbers and morphological references, Table S2: Primers and PCR settings for COI, 16S rDNA and 28S rDNA, Table S3: Pairwise distances of COI between species, Table S4: Pairwise distances of COI between the specimens of Clade 3.
