# Supplementary material for: Phylogeny and Cryptic Diversity of Diopatra (Onuphidae, Annelida) in the East Atlantic
Source: Biology (Basel). 2022 Feb 18;11(2):327. doi: 10.3390/biology11020327 (PMC8869602; doi:10.3390/biology11020327)
Supplement: Supplementary file 1 [file biology-11-00327-s001.zip › biology-1544771-supplementary/Supplementary Figures and Tables/Supplementary figure captions.pdf]

Figures S5-S8 can be found in the worksheets of the excel file “Supplementary Tables 1-4, Figures 5-8”

Figure S1. Consensus tree from the Bayesian analysis of the combined COI and 16S dataset; numbers on nodes indicate Bayesian posterior probabilities. Terminal names are specimen codes (column M in Table S1).

Figure S2. Consensus tree from the Bayesian analysis of 28S dataset; numbers on nodes indicate Bayesian posterior probabilities. Terminal names are specimen codes (column M in Table S1)

Figure S3. Consensus tree from the Bayesian analysis of 16S dataset; numbers on nodes indicate Bayesian posterior probabilities. Terminal names are specimen codes (column M in Table S1)

Figure S4. Consensus tree from the Bayesian analysis of COI dataset; numbers on nodes indicate Bayesian posterior probabilities. Terminal names are specimen codes (column M in Table S1)

Figure S5. bPTP species delimitation tree built based on COI dataset. Red clades represent putative species. Numbers on each node are posterior probabilities of the inner taxa forming one species. Terminal names are specimen codes (column M in Table S1)

Figure S6. bPTP species delimitation tree built based on 16S dataset. Red clades represent putative species. Numbers on each node are posterior probabilities of the inner taxa forming one species. Terminal names are specimen codes (column M in Table S1)

Figure S7. bPTP species delimitation tree built based on the combined COI and 16S dataset. Red clades represent putative species. Numbers on each node are posterior probabilities of the inner taxa forming one species. Terminal names are specimen codes (column M in Table S1)

Figure S8. bPTP species delimitation tree built based on 28S dataset. Red clades represent putative species. Numbers on each node are posterior probabilities of the inner taxa forming one species. Terminal names are specimen codes (column M in Table S1)

Figure S9. Posterior trace plot showing lack of convergence in MCMC run in bPTP analysis of 28S dataset.
