# Supplementary figures and images for: Phylogeny and Cryptic Diversity of Diopatra (Onuphidae, Annelida) in the East Atlantic
Source: Biology (Basel). 2022 Feb 18;11(2):327. doi: 10.3390/biology11020327 (PMC8869602; doi:10.3390/biology11020327)

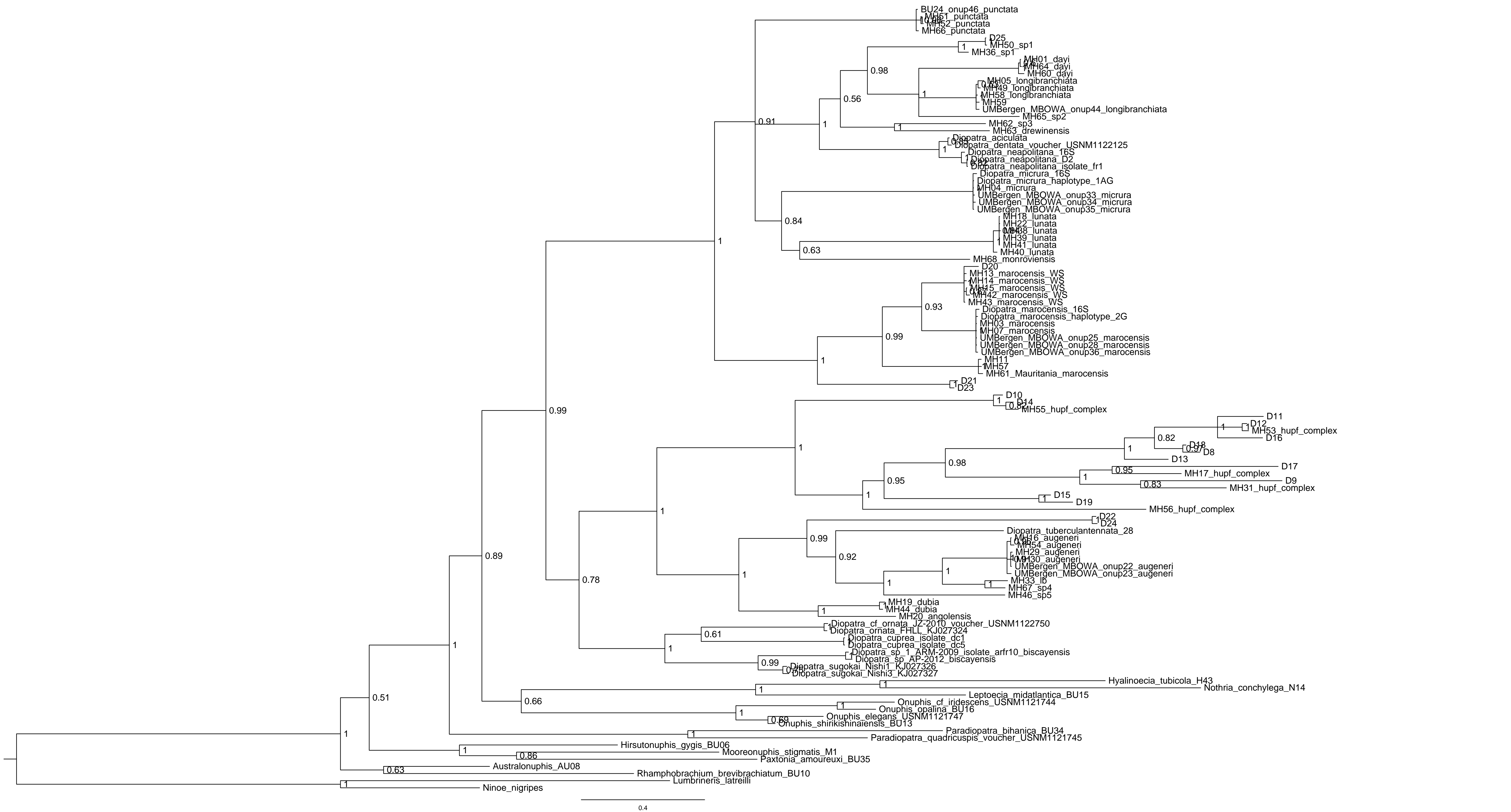

Supplement: Supplementary file 1 [file biology-11-00327-s001.zip › biology-1544771-supplementary/Supplementary Figures and Tables/Figure_S1_COI_16S_tree.pdf]

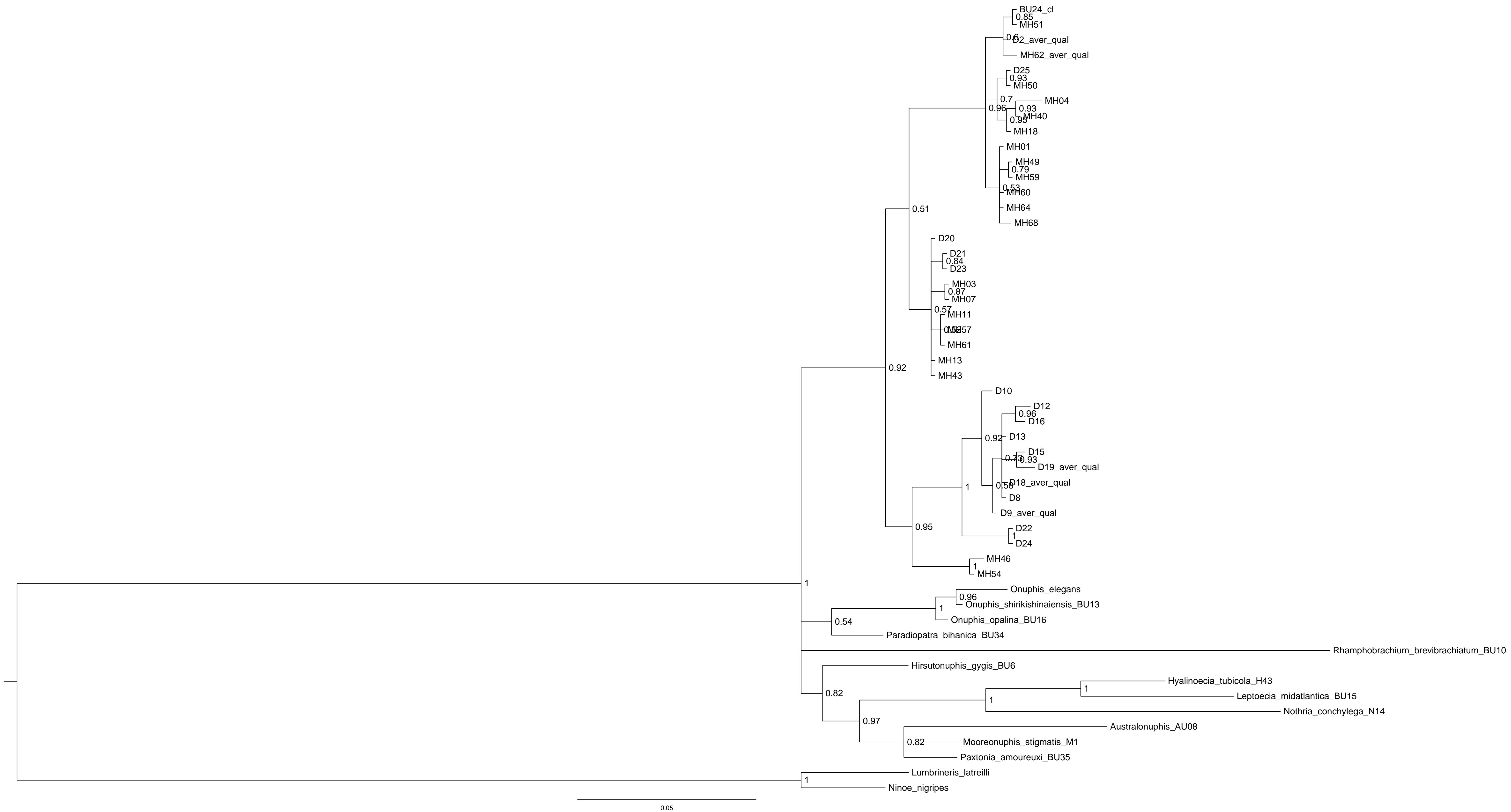

Supplement: Supplementary file 1 [file biology-11-00327-s001.zip › biology-1544771-supplementary/Supplementary Figures and Tables/Figure_S2_28S_tree.pdf]

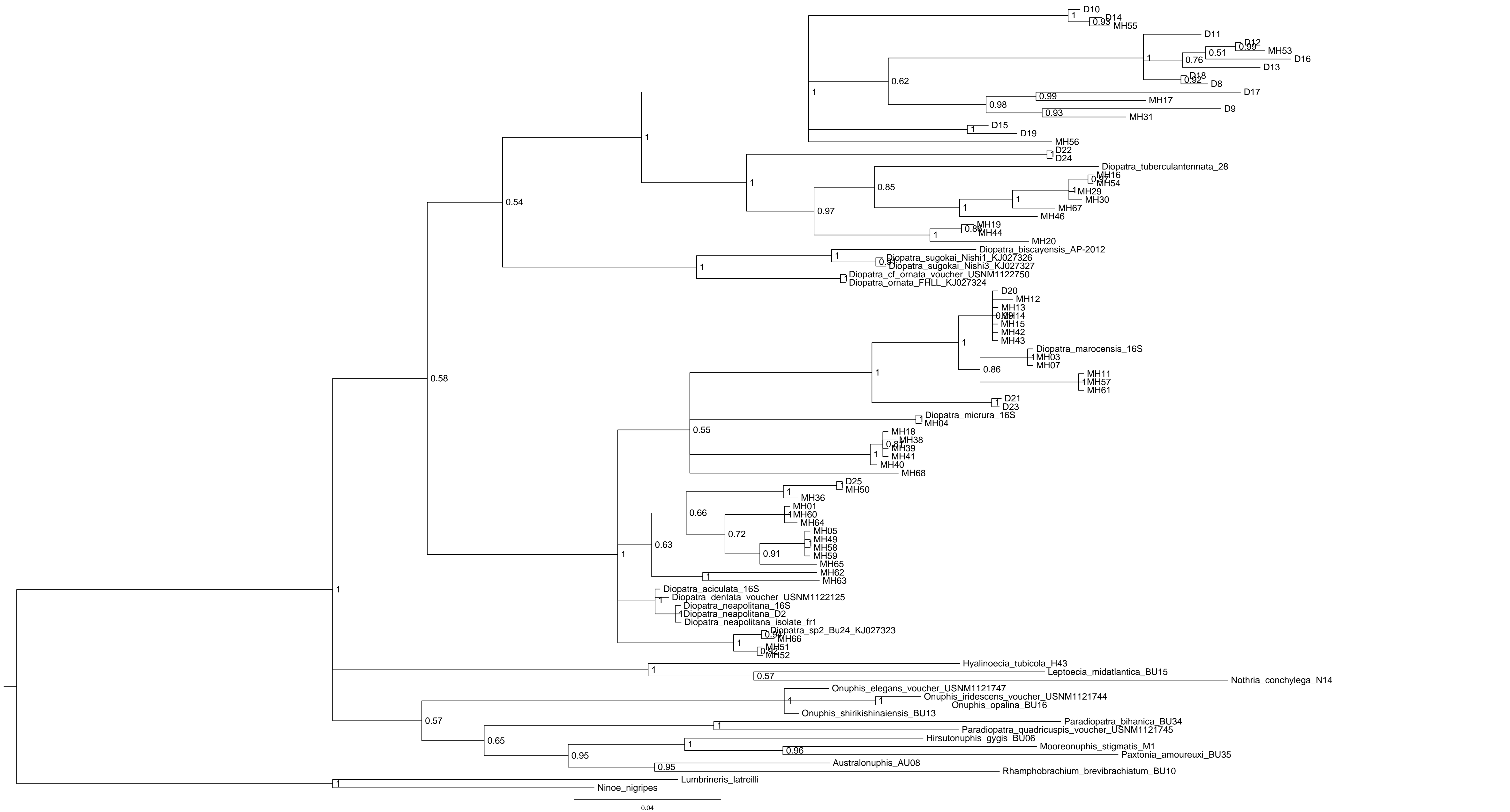

Supplement: Supplementary file 1 [file biology-11-00327-s001.zip › biology-1544771-supplementary/Supplementary Figures and Tables/Figure_S3_16S_tree.pdf]

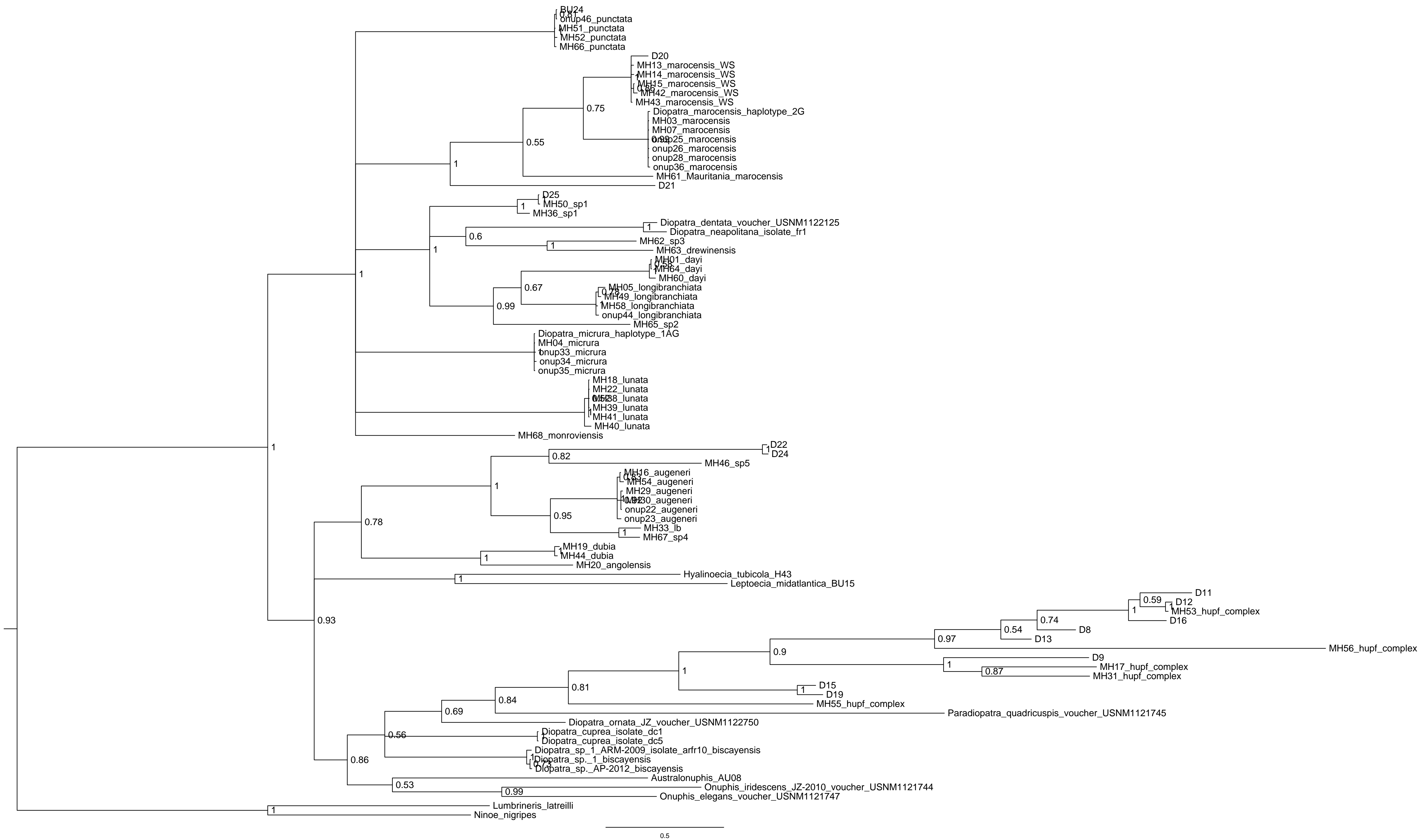

Supplement: Supplementary file 1 [file biology-11-00327-s001.zip › biology-1544771-supplementary/Supplementary Figures and Tables/Figure_S4_COI_tree.pdf]

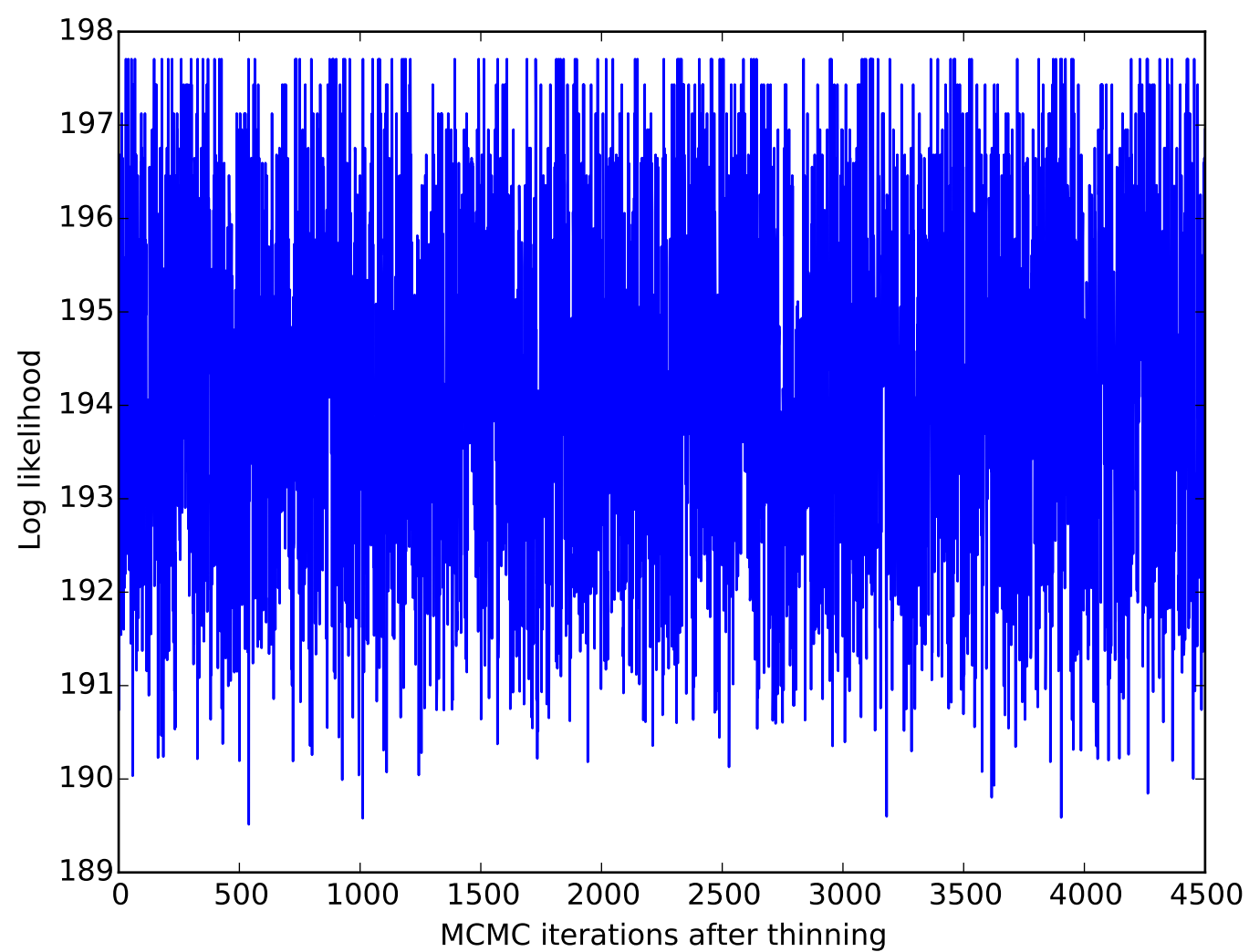

Supplement: Supplementary file 1 [file biology-11-00327-s001.zip › biology-1544771-supplementary/Supplementary Figures and Tables/Figure_S9_28s_trace.pdf]
